# Enhanced heart sound anomaly detection via WCOS: a semi-supervised framework integrating wavelet, autoencoder and SVM

**DOI:** 10.3389/fninf.2025.1530047

**Published:** 2025-01-29

**Authors:** Peipei Zeng, Shuimiao Kang, Fan Fan, Jiyuan Liu

**Affiliations:** ^1^Civil Aviation University of China Engineering Technology Training Center, Civil Aviation University of China, Tianjin, China; ^2^Aviation Ground Special Equipment Research Base of Civil Aviation Administration of China, Civil Aviation University of China, Tianjin, China; ^3^College of Safety Science and Engineering, Civil Aviation University of China, Tianjin, China; ^4^Pediatric Cardiac Center, Beijing Children’s Hospital, Capital Medical University, Beijing, China; ^5^College of Aeronautical Engineering, Civil Aviation University of China, Tianjin, China

**Keywords:** heart sound detection, semi-supervised anomaly detection, sample imbalance, convolutional autoencoder, one classification support vector machine

## Abstract

Anomaly detection is a typical binary classification problem under the condition of unbalanced samples, which has been widely used in various fields of data mining. For example, it can help detect heart murmurs when the heart is structurally abnormal, to tell if a newborn has congenital heart disease. Due to the low time and high efficiency, most work focuses on the semi- supervised anomaly detection method. However, the anomaly detection effect of this method is not high because of massive data with uneven samples and different noise. To improve the accuracy of anomaly detection under unbalanced sample conditions, we propose a new semi-supervised anomaly detection method (WCOS) based on semi-supervised clustering, which combines wavelet reconstruction, convolutional autoencoder, and one classification support vector machine. In this way, we can not only distinguish a small proportion of abnormal heart sounds in the huge data scale but also filter the noise through the noise reduction network, thus significantly improving the detection accuracy. In addition, we evaluated our method using real datasets. When the noise of sigma = 0.5, the AUC standard deviation of the WR-CAE-OCSVM is 19.2, 54.1, and 29.8% lower than that of WR-OCSVM, CAE-OCSVM and OCSVM, respectively. The results confirmed the higher accuracy of anomaly detection in WCOS compared to other state-of-the-art methods.

## 1 Introduction

With the change in the modern medical model, the spectrum of human diseases and death has undergone great changes, and birth defects have gradually become the main cause threatening children’s health, congenital heart disease is the most common type of birth defect disease, accounting for about 28% of all congenital malformations. Among the fatal defects in children under 5 years old, congenital heart disease is the first (Xue et al., 2022). Therefore, early and accurate diagnosis of congenital heart disease will make children get timely diagnosis and treatment, and significantly improve the prognosis of children. Cardiac auscultation, as a convenient and non-invasive examination method, is the most important means of early screening for congenital heart disease, and the accuracy of the results of auscultation has become an important factor affecting the screening effect of congenital heart disease. The traditional auscultation method requires the use of a general stethoscope by an audiologist with certain audiological skills and experience, but because of the high degree of subjectivity, the lack of audiological skills and experience of primary care doctors is often a bottleneck limiting the effectiveness of congenital heart disease screening. With the development of artificial intelligence technology, the collection of heart sounds by electronic stethoscope and the recognition of digital auscultation data by artificial intelligence algorithm make the artificial intelligence of cardiac auscultation possible (Wang et al., 2024).

Artificial intelligence auscultation will effectively assist doctors in judging the condition, greatly reduce the work intensity of doctors, and improve the accuracy of auscultation. Heart sound is a complex sound produced by the switching of heart valves, the relaxation and contraction of tendons and muscles, the impact of blood flow, and the vibration of the cardiovascular wall. Abnormal blood shunt will occur when the heart structure is abnormal, and then the heart murmur will be generated. Therefore, the detection of abnormal heart sounds is becoming an increasingly important research field (Jyothi and Pradeepini, 2021).

Abnormal detection will have different degrees of detection difficulties according to different samples and different processes of signal acquisition. First, for heart sound samples, sample imbalance will cause the model to be biased toward most normal samples, thus placing a higher emphasis on most normal samples in prediction, which may lead to the improvement of the accuracy of most normal samples and the decrease of the accuracy of a few abnormal samples. This bias makes it impossible for the model to accurately distinguish abnormal heart sound samples because abnormal samples usually belong to a small number of abnormal samples (Liang et al., 2021). At the same time, the heart sound signal is easily interfered with by various noises in the acquisition process, such as power line frequency noise, baseline drift, myoelectric interference (Xiao-dong et al., 2020; Shen et al., 2021), etc. These noises will reduce the quality of the heart sound signal and lead to the loss of information, which will affect the accuracy of the subsequent heart sound signal analysis and processing. Consequently, sample imbalance will cause the model to bias most normal classes and reduce the prediction accuracy of a few abnormal classes, while noise interference will reduce the robustness and feature learning ability of the model.

Effective identification of normal and abnormal heart sounds is a difficulty in artificial intelligence auscultation research (Ling et al., 2003), and it is urgent to establish an anomaly detection technology with a better detection effect to reduce the influence of sample heterogeneity and noise on detecting abnormal heart sounds. Deep learning technology has made a lot of progress in the field of anomaly detection, which involves a variety of deep neural network structures, loss functions, and optimization algorithms (Chong et al., 2021), including anomaly detection based on autoencoders, anomaly detection based on graph neural networks and anomaly detection based on deep generation models. Below is a detailed summary of the methods for detecting heart sound anomalies based on deep learning techniques.

## 2 Literature review of anomaly detection in heart sounds

The autoencoder is an unsupervised deep neural network that learns a compressed representation from the input data and reconstructs an output that is as similar as possible to the original data. Abnormal data usually cannot be reconstructed well, so the autoencoder can be used for anomaly detection. Recent studies have also combined other deep-learning techniques with autoencoders, such as autoencoders (Chen et al., 2024), variational autoencoders (Gangloff et al., 2024), and generative adversarial networks (Ruff et al., 2021; Li, 2024). The disadvantage is that the autoencoder may learn abnormal data features and thus reduce the detection accuracy, and the reconstruction error cannot accurately reflect the abnormal degree of the data.

Graph neural network is a kind of deep learning model specially used to process graph-structured data (Mir et al., 2023). Abnormal data is usually some unusual node or edge in the data, so you can translate the anomaly detection problem into detecting abnormal nodes or edges in the graph structure. The latest research shows that the anomaly detection method based on graph neural networks has achieved very good results in the heart sound signal recognition scene (Rezaee et al., 2022) and a variety of other scenes (Niu et al., 2021). To alleviate the nature and scalability of the scene, combined with other methods, variational graph convolutional networks (Mir et al., 2024), two-domain graph convolutional networks (Li et al., 2023), space–time graph networks (Yuan et al., 2024), and so on are proposed. The disadvantage is that the computational complexity of the graph neural network is high, which requires more computational resources, and the model is highly dependent on the accuracy and integrity of the graph structure, which affects the anomaly detection effect.

Deep generation models are a class of deep learning models that can learn a probability distribution from data and generate new data similar to the original data. The latest research shows that the anomaly detection method based on the deep generation model has high flexibility and robustness, and can be applied to various types of data, such as text data (Fan et al., 2021), image data (Sanders et al., 2020), and time series data (Gao et al., 2021).

There are also other methods based on mathematical models, such as establishing an indiscernibility-assisted intuitionistic fuzzy-rough set model based on fuzzy and rough set theories to reduce the noise (Shreevastava et al., 2023)^,^ establishing a missing value estimation and feature selection method to reduce the dimensionality while maintaining the performance (Jain et al., 2023), integrating the fuzzification module and the RBFNN, designing an adaptive control scheme to effectively reduce the model’s detection uncertainty (Kumar et al., 2024), and designing an adaptive control scheme based on intuitionistic fuzzy interference to resist noise and better handle uncertainty in judgment and recognition (Jain et al., 2022). The shortcomings of the mathematical model-based approach are that the model efficacy depends on the data quality and distribution, the parameter tuning is challenging, and the generalization to different datasets needs to be further investigated.

To sum up, anomaly detection based on autoencoders performs well in terms of simplicity and generalization ability but lacks in terms of sensitivity to abnormal data and reconstruction error for heart sound data (Cloudera and Nisha, 2020). Anomaly detection based on graph neural networks performs well in handling complex relationships and unifying frameworks, but it is highly computation-based and depends on graph structure (Jia-Yan et al., 2020). However, anomaly detection based on a depth generation model has significant advantages in terms of representation ability, automatic feature extraction, and wide applicability of cardiac sound anomaly data (Xing et al., 2020). Therefore, based on the deep generation model and inspired by the DCGAN network architecture model (Liu et al., 2019) and the semi-supervised value detection framework (Shi et al., 2023), this study intends to collect the heart sound data of children in our center. Combined with the semi-supervised anomaly detection framework of wavelet reconstruction (Aziz et al., 2024), convolutional autoencoder (Li et al., 2024), and one classification support vector machine (Zhang et al., 2025), a method for differential analysis of normal heart sounds and abnormal heart sounds was constructed and verified, the name of the framework is WCOS, which is short for WR-CAE-OCSVM. The research results will provide a reference for further research on the classification of heart noises.

The subsequent chapters of this paper are arranged as follows. Firstly, Chapter 2 describes the framework construction process, focusing on wavelet reconstruction and convolutional autoencoder. Secondly, as an application verification, Chapter 3 uses real data to verify the validity of the proposed model. Finally, in Chapter 4, we give the main conclusions of the paper and future research work.

The subsequent chapters of this paper are organized as follows. Firstly, the opening of Chapter 2 describes the key significance of heart sound abnormality detection in the diagnosis of congenital heart disease and puts forward the difficulties of sample imbalance and noise interference, followed by Chapter 3, which comprehensively analyzes the limitations of traditional means and the advantages and disadvantages of each method of deep learning, and analyzes the performances of a variety of models, and Chapter 4, which describes the process of constructing the framework of the WCOS model and analyzes the principles of noise reduction by wavelet, convolutional self-encoder feature mining, and classification by support vector machine. In Chapter 5, the validity of the proposed model is verified based on heart sound signals collected by an electronic stethoscope. Finally, in Chapter 6, we give the main conclusions of the article as well as an analysis of the model’s strengths and weaknesses.

## 3 Theory of the developed method

In this paper, we organically combine the wavelet reconstruction (WR), the convolutional auto-encoder (CAE), and one classification support vector machine (OCSVM) to construct a new semi-supervised exception detection framework. This section first introduces the overall process of the proposed framework and briefly introduces the training and testing process of the framework. Then, we introduce the important components of the framework: wavelet reconstruction, convolutional autoencoder, and one classification support vector machine.

### 3.1 Fundamental architectures of the WCOS model

**Figure 1:**
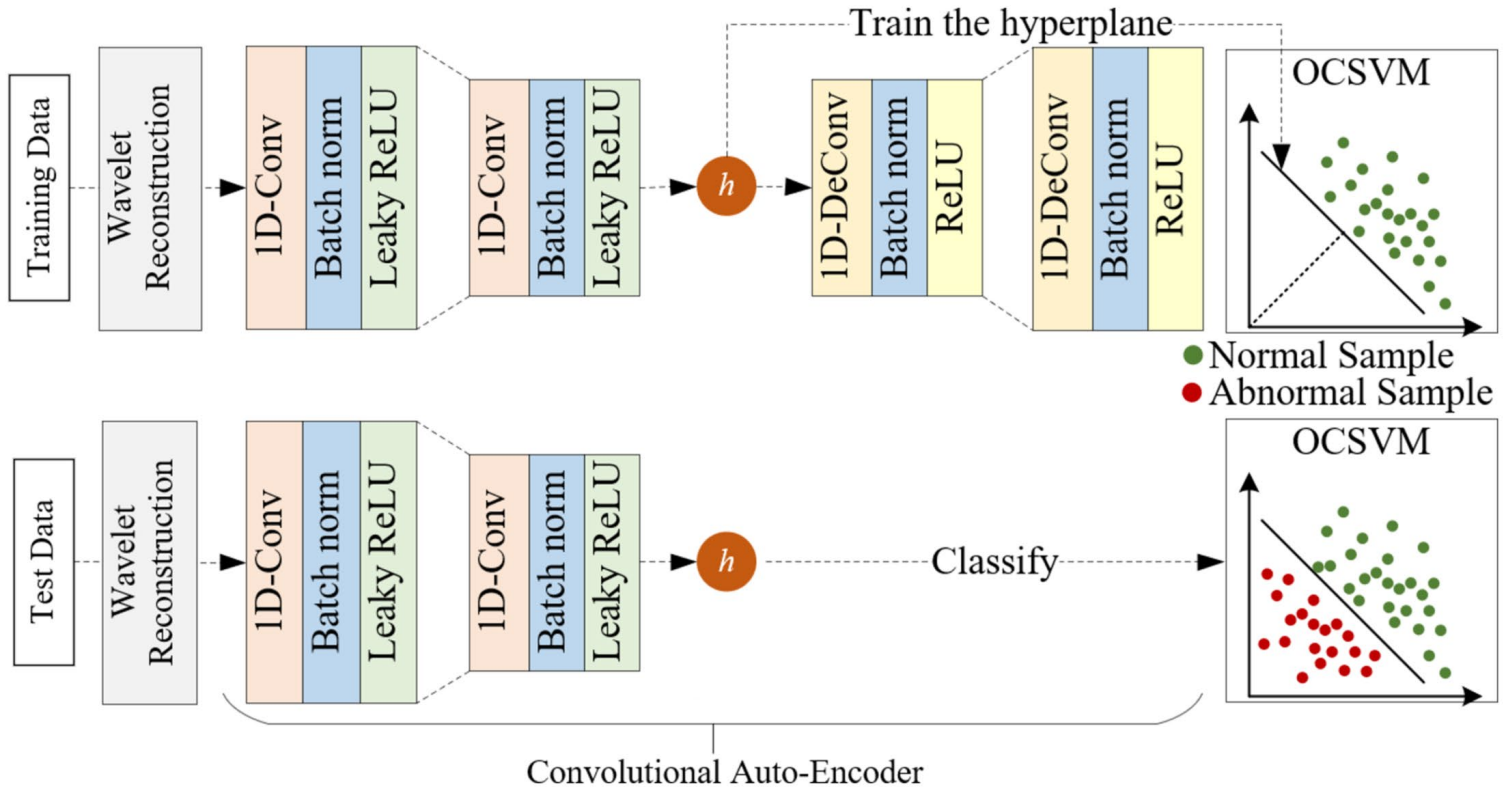
Overall framework of the proposed WCOS model.

The overall framework of WCOS proposed in this paper mainly includes three parts: wavelet reconstruction, convolutional autoencoder, and one classification support vector machine, as shown in Figure 1.

The upper part of Figure 1 shows the training process of the WCOS model. Firstly, the original signal is decomposed by a multilayer wavelet, the high-frequency information containing noise is zeroed, and then the low-frequency information is reconstructed to obtain the reconstructed signal. Then, the reconstructed signals are divided by the sliding window method to obtain one sample after another. Finally, the training set and the test set are divided by the method of five-fold crossover. After partitioning the data set, the WCOS model is trained. The training steps of the model are summarized as follows: (A) Train the convolutional autoencoder using normal samples, the loss function – reconstruction error is minimized by backpropagation algorithm; (B) Train the one classification support vector machine using the potential representation of normal samples, minimize the loss function.

The bottom section of Figure 1 shows the testing process for the WCOS model. The testing steps of the model are summarized as follows: (A) Input the test sample into the convolutional autoencoder and obtain the corresponding latent representation; (B) Input the potential representation of the sample into the one classification support vector machine and obtain the corresponding anomaly score. Finally, the abnormal score was used to diagnose patients with cardiac abnormalities.

Next, the important components of the model are described in detail: wavelet reconstruction, convolutional self-encoder, and one-classification support vector machine.

### 3.2 Wavelet reconstruction

**Figure 2:**
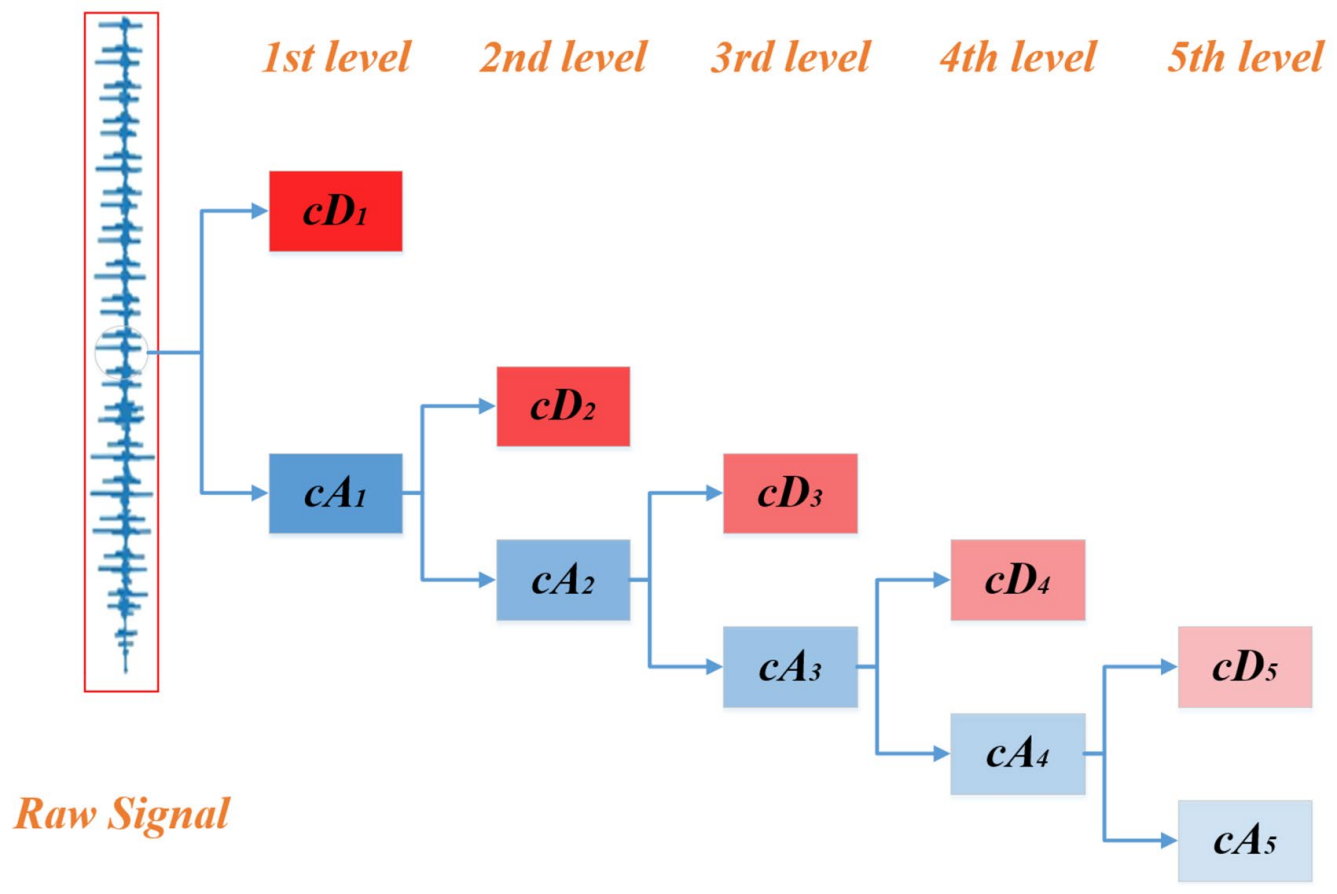
Wavelet tree of the raw signal.

Wavelet reconstruction is used to denoise heart sounds. As a classical time-scale analysis algorithm, wavelet transform can decompose the signal into two sets of wavelet coefficients: approximation coefficient and detail coefficient. As shown in Figure 2, the approximate coefficients can be decomposed recursively, allowing for a more “detailed” examination of the original signal. In general, the approximate coefficient represents the information of the low-frequency part of the signal and is considered as the trend term of the signal. The detail coefficient characterizes the information of the high-frequency part of the signal, which is considered as the noise term of the signal. Therefore, to avoid the influence of noise, it is necessary to retain the approximate coefficient and discard the detail coefficient in the process of reconstruction.

In practical engineering, a series of low-pass filters and high-pass filters are usually used for discrete wavelet transform. Specifically, the wavelet decomposition formula for the original signal 𝑥 is shown as follows function of Equation 1:

(1) $$\left\{ \begin{array}{l} cA\left(k\right)=\sum x\left(n\right)h\left(2k-n\right)\\ cD\left(k\right)=\sum x\left(n\right)g\left(2k-n\right) \end{array}\right.$$

where $x\left(n\right)$ denotes the sampled value of the original signal at discrete moments, $cA\left(k\right)$ denotes the approximation coefficient of the signal, $cD\left(k\right)$ denotes the detail coefficient, $h\left(\cdot \right)$ denotes the low-pass filter, $g\left(\cdot \right)$ denotes the high-pass filter, k is the index of the coefficients after the discrete wavelet transform, n is the sampling index of the original signal.

Wavelet reconstruction is the inverse operation of wavelet decomposition, and the formula of wavelet reconstruction is as follows function of Equation 2:

(2) $$x\left(k\right)=\underset{n}{\sum }cA\left(n\right)h\left(k-2n\right)+\underset{n}{\sum }cD\left(n\right)g\left(k-2n\right)$$

Where $x\left(k\right)$ denotes the sampled value of the reconstructed signal at discrete moments, $cA\left(n\right)$ , $cD\left(n\right)$ . Same meaning as in wavelet decomposition, approximation coefficients and detail coefficients, respectively, but here as inputs to the reconstruction, $\left(k-2n\right)$ , $g\left(k-2n\right)$ still represent low-pass and high-pass filters, respectively, but the indexing is different here and is the operation used to reconstruct the original signal from the coefficients.

To avoid noise, set the detail parameters $cD\left(n\right)$ of the above equation to zero, then the above formula becomes the function of Equation 3 below:

(3) $$x\left(k\right)=\underset{n}{\sum }cA\left(n\right)h\left(k-2n\right)$$

Where, $cA\left(n\right)$ denotes the approximation coefficient of the signal. This removes the high-frequency noise component of the signal, which tends to contain more noise, while the low-frequency component retains the main information of the signal. At this time, the reconstructed signal not only retains the main information of the original signal but also avoids the influence of noise to a certain extent, so the reconstructed signal is more suitable for the detection of cardiac abnormalities.

The Symlet 4 (sym4) wavelet was used in this study for wavelet decomposition. The sym4 wavelet was chosen because it provides a favorable trade-off between time and frequency localization of heart sound signals. The complexity of heart sound waveforms, which are often disturbed by noise, calls for a wavelet that can accurately decompose both low-frequency fundamental rhythms as well as high-frequency transient events such as murmurs. sym4 wavelet’s design features make it capable of such tasks. Its symmetrical nature reduces phase distortion during decomposition and reconstruction, which is essential for maintaining the integrity of the signal’s phase information, which is critical for recognizing subtle differences in heartbeat patterns.

Regarding the level of wavelet decomposition, a 5-level decomposition was used. The five-level decomposition provides a fine-grained exploration of the signal spectrum. The initial levels capture the broad low-frequency trends that underpin the normal cardiac cycle. As the level of decomposition increases, details and potential anomalies in the high-frequency range gradually become apparent. For example, subtle changes in murmur intensity and frequency can be better separated and characterized at these higher levels. Thus, this five-level approach allows the extraction of discriminative features from different frequency layers and improves the efficiency of subsequent anomaly detection procedures by providing a comprehensive spectral depiction of the heart sound signal.

### 3.3 1-D convolutional auto-encoder

Convolutional autoencoders are used to extract the characteristic information of heart sounds. The convolutional autoencoder consists of an encoder and a decoder. The special feature is that the convolutional autoencoder uses a convolutional layer to replace the fully connected layer in the encoder, and a deconvolution layer to replace the fully connected layer in the decoder. The specific structure of the convolutional autoencoder in this paper is shown in Figure 1: the encoder consists of two convolutional layers, a batch layer, and a Leaky ReLU activation function, while the decoder consists of two deconvolution layers, a batch layer, and a ReLU activation function, and finally a Tanh layer.

The convolutional layer is an important component of convolutional autoencoders, which can greatly reduce the number of parameters in the network. On the one hand, it can improve the robustness of the network; On the other hand, it can also reduce the risk of network overfitting. For the single-channel input 𝑥, the potential representation of the 𝑖 feature map is formally defined as follows Equation 4:

(4) $$h^{i}=f\left(x⊗W^{i}+B^{i}\right)$$

Where f represents the activation function in the encoder (we use Leaky ReLU in this article), $B^{i}$ represents the bias of the 𝑖 feature map, and the symbol ⊗ represents the two-dimensional convolution operation.

The deconvolution layer can be seen as an inverse mapping of the convolution layer, and the reconstructed representation for the input 𝑥 is defined as follows Equation 5:

(5) $$\hat{x}=g\left(\underset{i\in H}{\sum }h^{i}⊗{\hat{W}}^{i}+c\right)$$

Where *g* represents the activation function in the decoder (ReLU is used in this article), 𝑐 represents the bias of each input channel, and *H* represents a set of potential feature maps.

In addition, the loss function of the convolutional autoencoder is usually the reconstruction error of the sample, which can be expressed as follows Equation 6 below:

(6) $$L_{cae}=\frac{1}{\left|x\right|}\underset{x\in X}{\sum }||x-\hat{x}||{}_{1}$$

Where the $||\cdot ||{}_{1}$ represents the 1-norm. 1-norm can obtain a clearer reconstructed sample than 2-norm, so this paper uses 1-norm to measure the reconstruction error between the input sample and the reconstructed sample.

### 3.4 One-class support vector machine

**Figure 3:**
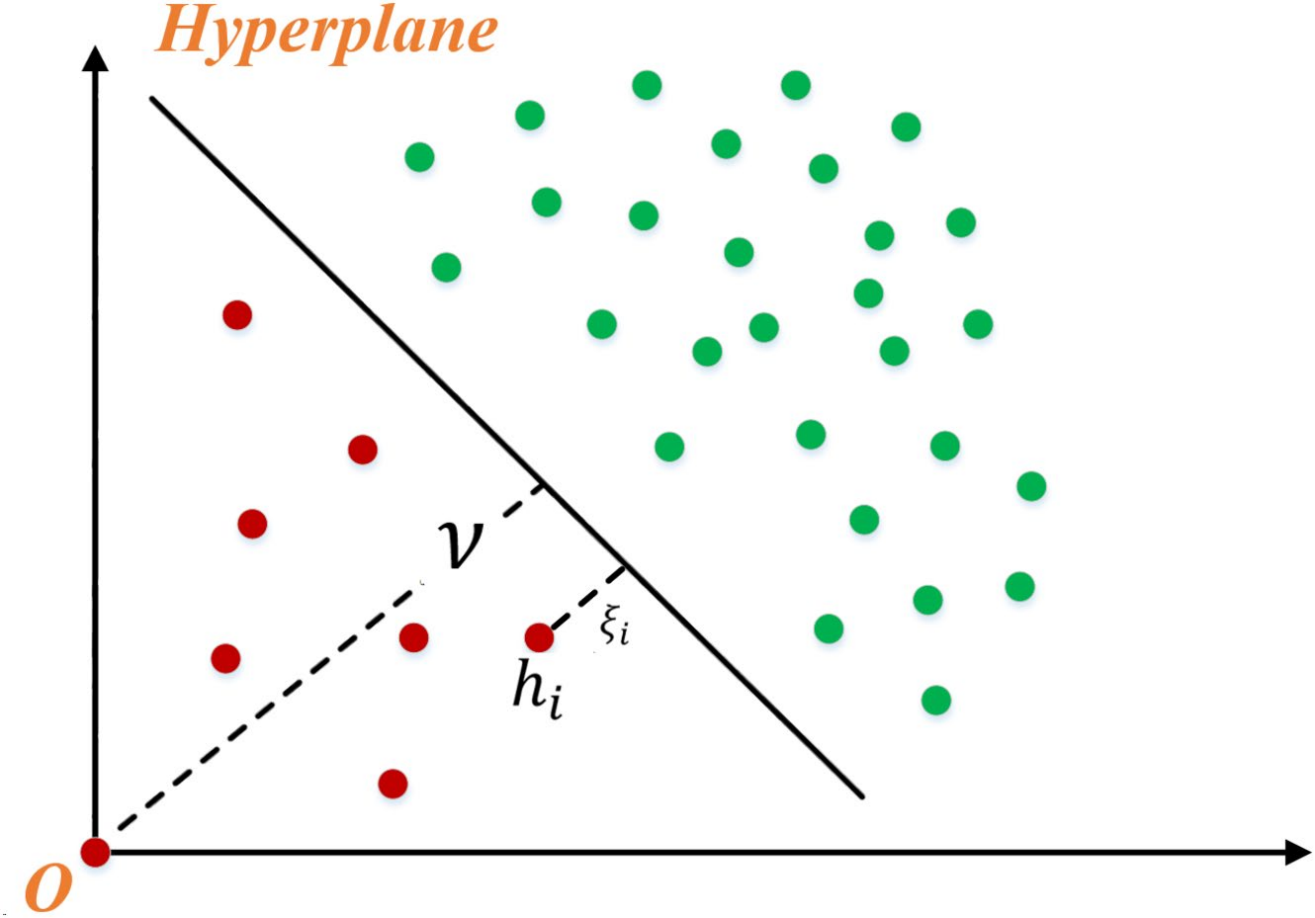
Sketch of one-class support vector machine.

OCSVM is used to diagnose heart sounds based on characteristic information. After the potential characteristics of samples are obtained by CAE, the boundary of normal samples needs to be learned to distinguish normal samples from abnormal samples. As a classical semi-supervised learning algorithm, the one classification SVM only uses normal samples for training, so compared with supervised algorithms, it can avoid the training problems caused by the high imbalance between normal samples and abnormal samples. As shown in Figure 3, the basic principle of OCSVM is to map the normal sample into a high-dimensional space through the kernel function, treat the origin as the only outlier, learn an optimal hyperplane (with maximum spacing between the origin and the normal sample), and can distinguish the origin from the normal sample.

For a given data set $H=\left\{h_{i}:i=1,2.\cdots N\right\}$ and the features of the data mapped to high-dimensional space mapping function $\varphi \left(\cdot \right)$ , where $\varphi \left(\cdot \right)$ can be computed by the kernel function of Equation 7:

(7) $$k\left(h_{i},h_{j}\right)=\varphi {\left(h_{i}\right)}^{T}\varphi \left(h_{j}\right)$$

where $h_{i}$ and $h_{j}$ are two data points and $\varphi \left(\cdot \right)$ is the feature mapping function. To make data set *H* away from the origin, equivalent to the following optimization problem Equation 8:

(8) $$\begin{array}{l} \min_{w,\xi ,v}\frac{1}{2}||w||{}^{2}+\frac{1}{CN}\underset{i}{\sum }\xi_{i}-v\\ \mathrm{subjectto}\left\{\begin{array}{l} \left(w\cdot \varphi \left(h_{i}\right)\right)\geq v-\xi_{i}\\ \xi_{i}\geq 0 \end{array}\right. \end{array}$$

where 𝑤 represents a vector perpendicular to the hyperplane; $\xi_{i}$ represents the relaxation variable, which can solve the outlier in the training set. *v* is the distance to the origin; C∈(0,1] represents the control factor that controls the complexity of the model, N is the total number of samples in the dataset H , subject to denote the constraints, and $\varphi \left(\cdot \right)$ is the previous kernel function formula. It is worth noting that in this paper the dataset 𝐻 consists of potential representations of samples.

After solving the above optimization problem, to obtain the weight of a set of models $\left\{w_{i}:i=1,2,\cdots ,N\right\}$ , for any of the test sample $h_{p}$ , its corresponding decision function is Equation 9 below:

(9) $$f\left(h_{p}\right)=sgn\left(\underset{i}{\sum }w_{i}k\left(h_{p},h_{i}\right)-v\right)$$

Where $sgn\left(\cdot \right)$ represents a symbolic function. If the decision function $f\left(h_{p}\right)=-1$ of sample $h_{p}$ is tested, then sample $h_{p}$ is diagnosed as normal. Conversely, if the decision function of test sample $h_{p}$ , then test sample $h_{p}$ is diagnosed as an abnormal sample. Thus anomaly detection under unbalanced conditions can be realized.

### 3.5 Evaluation metrics of the WCOS model

The Area Under the Curve (*AUC*) of Receiver Operating Characteristics (*ROC*) was used as the evaluation index, *ROC* abscissa is the false positive rate (FPR), the ordinate is the true positive rate (TPR), and the area surrounded by the coordinate axis is defined as *AUC*. Studies show that *AUC* can better measure the performance of the classifier than the overall accuracy under the condition of uneven data. *AUC* is an index to evaluate the quality of the binary classification model, given by Equation 10:

(10) $$\begin{array}{l} AUC=\frac{\sum_{i\in \mathrm{positionClass}}\mathrm{ran}k_{i}-\frac{M\left(1+M\right)}{2}}{M\times N}\\ \underset{i\in \mathrm{positionClass}}{\sum }\mathrm{ran}k_{i} \end{array}$$

where *positionClass* represents the positive set, $\mathrm{ran}k_{i}$ represents the rank of the *ith* sample in the sample ranking, and the $\underset{i\in \mathrm{positionClass}}{\sum }\mathrm{ran}k_{i}$ term represents the sum of the ranks belonging to the positive samples. M and N represent True Positive Rate (TPR) and False Positive Rate (FPR), respectively.

## 4 Experimental results

### 4.1 Data set preparation and preprocessing

To verify the validity of the constructed heart sound abnormality detection model, a Littmann 3200 electronic stethoscope from 3 M Company, USA, was used to collect heart sound signals to construct the dataset. The data were processed using a 5-fold cross-validation method, and the data were derived from actual clinical measurements in multiple medical institutions, not from simulations. In clinical practice, abnormalities in cardiac structure or function are associated with changes in the rhythm, frequency, and intensity of heart sounds. For example, murmurs caused by valvular lesions and weakened or enhanced heart sounds due to myocardial lesions are selected as key indicators for the detection of abnormalities. At the same time, we searched patients’ medical records, diagnostic reports, and treatment feedback to accurately determine the status of heart sounds, constructed samples using sliding window technology, and analyzed a large number of cases to clarify that the length of abnormal heart sound sequences was mostly in the range of 6 to 10, and then set the sliding window size to 10.

This paper is experimentally verified on a device with a six-core Intel(R) Core (TM)i7-9750HCPU@2.59GHz processor and 8GB DDR4 memory.

### 4.2 Experimental setup

The original signal statistics method TF24 calculates the original signal through a series of specific formulas, and its role is to decomposition the original sound signal into 24 parameters, which cover a variety of characteristics of the signal in the time domain and frequency domain. The main purpose of TF24 is to extract representative features from the original signal, which can describe the characteristics of the original signal more comprehensively. The usage of TF24 is to use its 24 extracted parameters as features as the input of subsequent models (such as OCSVM). The time domain parameters are expressed as p1 ~ p11 and given by Equation 11:

(11) $$\left\{\begin{array}{cc} p_{1}=\frac{\sum_{n=1}^{N}x\left(n\right)}{N} & p_{2}=\sqrt{\frac{\sum_{n=1}^{N}{\left(x\left(n\right)-p_{1}\right)}^{2}}{N-1}}\\ p_{3}={\left(\frac{\sum_{n=1}^{N}\sqrt{\left|x\left(n\right)\right|}}{N}\right)}^{2} & p_{4}=\sqrt{\frac{\sum_{n=1}^{N}{\left(x\left(n\right)\right)}^{2}}{N}}\\ p_{5}=\max\left|x\left(n\right)\right| & p_{6}=\frac{\sum_{n=1}^{N}{\left(x\left(n\right)-p_{1}\right)}^{3}}{\left(N-1\right)p_{2}^{3}}\\ p_{7}=\frac{\sum_{n=1}^{N}{\left(x\left(n\right)-p_{1}\right)}^{4}}{\left(N-1\right)p_{2}^{4}} & p_{8}=\frac{p_{5}}{p_{4}}\\ p_{9}=\frac{p_{5}}{p_{3}} & p_{10}=\frac{p_{4}}{\frac{1}{N}\sum_{n=1}^{N}\left|x\left(n\right)\right|}\\ p_{11}=\frac{p_{5}}{\frac{1}{N}\sum_{n=1}^{N}\left|x\left(n\right)\right|} & \end{array}\right.$$

In Equation 11, p1 represents the average amplitude of the signal in the time domain, p2 calculates the standard deviation of the signal in the time domain, p3 is related to some weighted summation of the signal amplitude, p4 represents the root mean square value of the signal in the time domain, p5 directly obtains the maximum amplitude of the signal in the time domain, p6–p11 These parameters involve higher order statistics of the signal amplitude relative to the mean.

In addition, the original signal statistical method decomposed the sound signal into 13 parameters in the time domain, expressed as p12 ~ p24, which is given by Equation 12:

(12) $$\left\{\begin{array}{cc} p_{12}=\frac{\sum_{k=1}^{K}s\left(k\right)}{K} & p_{13}=\frac{\sum_{k=1}^{K}{\left(s\left(k\right)-p_{12}\right)}^{2}}{K}\\ p_{14}=\frac{\sum_{k=1}^{K}{\left(s\left(k\right)-p_{12}\right)}^{3}}{K{\left(\sqrt{p_{13}}\right)}^{3}} & p_{15}=\frac{\sum_{k=1}^{K}{\left(s\left(k\right)-p_{12}\right)}^{4}}{Kp_{13}^{2}}\\ p_{16}=\frac{\sum_{k=1}^{K}f_{k}s\left(k\right)}{\sum_{k=1}^{K}s\left(k\right)} & p_{17}=\sqrt{\frac{\sum_{k=1}^{K}{\left(f_{k}-p_{16}\right)}^{3}s\left(k\right)}{K}}\\ p_{18}=\sqrt{\frac{\sum_{k=1}^{K}{f_{k}}^{2}s\left(k\right)}{\sum_{k=1}^{K}s\left(k\right)}} & p_{19}=\sqrt{\frac{\sum_{k=1}^{K}f_{k}^{4}s\left(k\right)}{\sum_{k=1}^{K}f_{k}^{2}s\left(k\right)}}\\ p_{20}=\frac{\sum_{k=1}^{K}{f_{k}}^{2}s\left(k\right)}{\sqrt{\sum_{k=1}^{K}f_{k}^{4}s\left(k\right)\sum_{k=1}^{K}s\left(k\right)}} & p_{21}=\frac{p_{17}}{p_{16}}\\ p_{22}=\frac{\sum_{k=1}^{K}{\left(f_{k}-p_{16}\right)}^{3}s\left(k\right)}{Kp_{17}^{2}} & p_{23}=\frac{\sum_{k=1}^{K}\sqrt{f_{k}-p_{16}}s\left(k\right)}{Kp_{17}^{4}}\\ p_{24}=\frac{\sum_{k=1}^{K}{\left(f_{k}-p_{16}\right)}^{4}s\left(k\right)}{K\sqrt{p_{17}}} & \end{array}\right.$$

In Equation 12, p12 represents the vibration energy in the frequency domain, and p13-p15, p17, and p21-p24 represent the convergence of the power spectrum. p16, p18, p19 and p20 represents the pattern of change of the main frequency.

The experiments involved four modules, namely OCSVM model, raw signal statistics TF24, convolutional autoencoder, and wavelet decomposition. Five models were set up for comparison, the control group is shown in Table 1.

**Table 1:** Control group setup.

Control group no	Control model structure
1	OCSVM Model
2	TF24-OCSVM
3	CAE-OCSVM
4	WR-OCSVM
5	WR-TF24-OCSVM
6	WR-CAE-OCSVM

As can be seen in the above table, the first group OCSVM represents a classification support vector machine, the second group TF24-OCSVM represents the combination of raw signal statistical method (TF24) and OCSVM, and the third group CAE-OCSVM represents the combination of convolutional autoencoder (CAE) and OCSVM. The fourth group of WR-OCSVM represents the combination of wavelet reconstruction (WR) and OCSVM, the fifth group of WR-TF24-OCSVM integrates wavelet reconstruction, raw signal statistical methods and OCSVM, and the sixth group of WR-CAE-OCSVM combines wavelet reconstruction, convolutional autoencoder and OCSVM.

### 4.3 Result analysis

#### 4.3.1 Comparison with other models

Comparing multiple current anomaly detection models, We chose the variant autoencoder (VAE)-based anomaly detection method, which is currently widely used and influential in heart sound anomaly detection or related fields, is selected as the comparison object. The data used in the comparison experiments are from the same source as those used to validate the WCOS model in the dissertation, and the software and hardware environments run are the same to ensure the consistency and comparability of the data.

In the following, the heart sound detection error of the VAE model will be verified first, and the mean square error (MSE) will be chosen as the evaluation index, as shown in the following formula Equation 13:

(13) $$MSE=\frac{1}{n}\overset{n}{\underset{i=1}{\sum }}{\left(y_{i}-{\dot{y}}_{i}\right)}^{2}$$

where n represents the number of samples, $y_{i}$ represents the true value of the i th sample, and ${\dot{y}}_{i}$ represents the predicted value of the i th sample.

**Figure 4:**
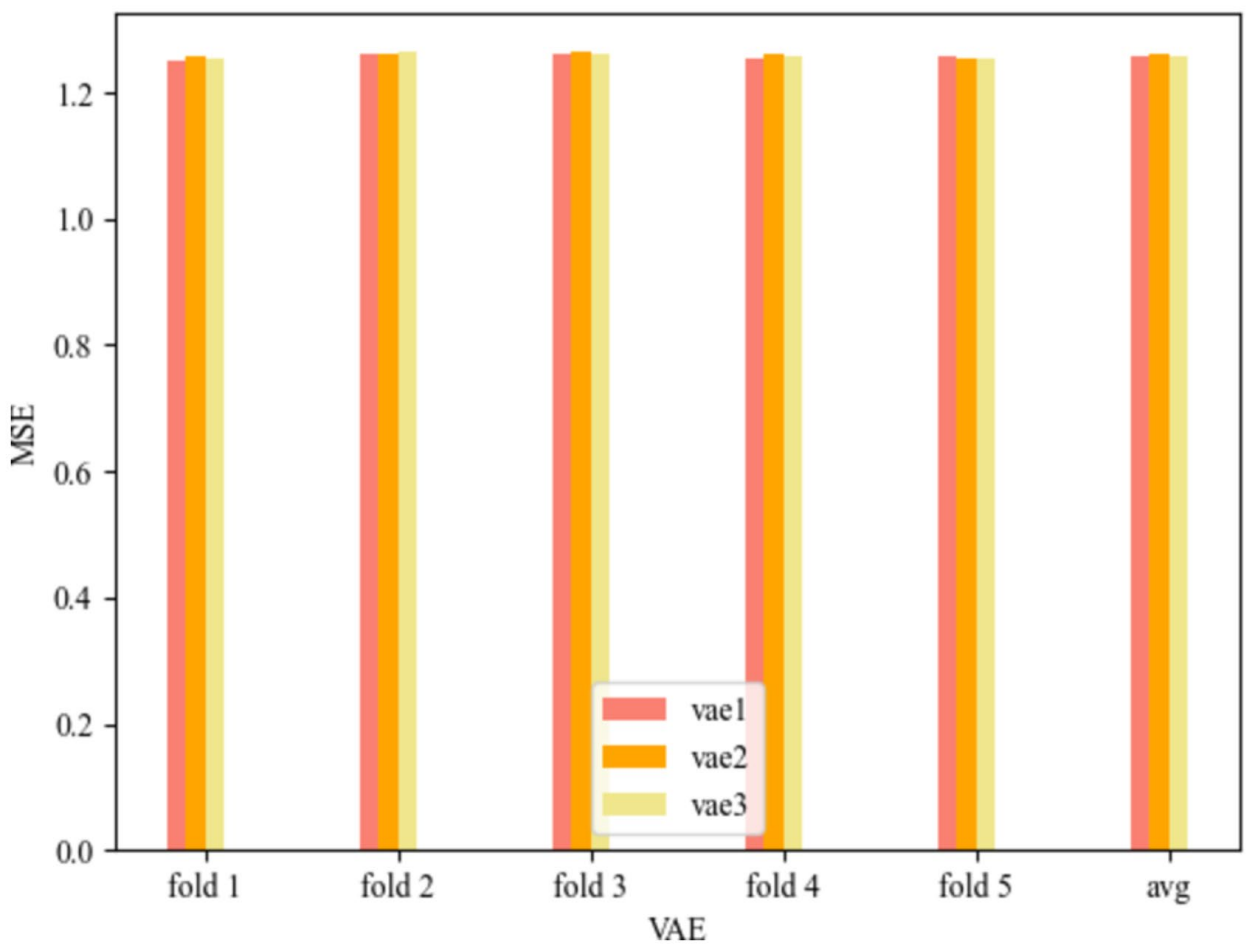
MSE of VAE.

The results of utilizing the five-fold method for the VAE model results are shown in Figure 4.

The VAE models for the three convolutional kernel cases can be seen in Figure 4. vae1 in Figure 4 represents the Test MSE for the VAE model with convolutional kernel of Wang et al. (2024) and Liang et al. (2021) is 1.257207727, vae2 represents the Test MSE for convolutional kernel of Liang et al. (2021) and Chong et al. (2021) is 1.259705973, and vae3 represents the Test MSE for convolutional kernel of Chong et al. (2021) and Mir et al. (2024) is 1.257824469. The smallest MSE is 1.257207727 when the number of convolutional kernels is (Wang et al., 2024; Liang et al., 2021). The maximum MSE is 1.259705973 when the number of convolution kernels is (Liang et al., 2021; Chong et al., 2021). The range of model MSE is during the range of [1.2572, 1.2597].

To compare with the WCOS model proposed in this paper, the root mean square error obtained by combining the VAE model with wavelet variations, a one-dimensional support vector machine, and using the five-fold method is shown below.

**Figure 5:**
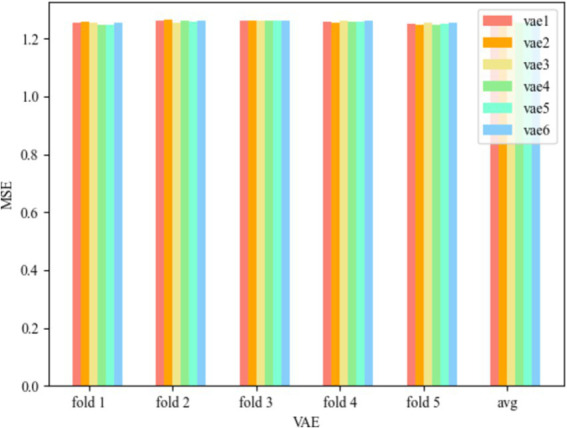
MSE of WR-VAE-OCSVM.

Figure 5 represents the MSE of WR-VAE-OCSVM for the six convolutional kernel cases, and the Test MSE of the VAE model with the convolutional kernel of Wang et al. (2024) and Liang et al. (2021) is 1.257626295, that of the convolutional kernel of Liang et al. (2021) and Chong et al. (2021) is 1.257779431, that of the convolutional kernel of Chong et al. (2021) and Mir et al. (2024) is 1.258005643, that of the convolutional kernel for Mir et al. (2024) and Li et al. (2024) has a Test MSE of 1.256320214, the Test MSE for convolution kernel for [32, 64] has a Test MSE of 1.256026459, and the Test MSE for convolution kernel for [64, 128] has a Test MSE of 1.259554124, and it can be seen that the model error is minimized at convolution kernel for [32, 64], when the Test MSE is 1.256026459, and when the convolution kernel is [64, 128], the model error is the largest, at this time the Test MSE is 1.259554124, and the error range is in [1.256026459, 1.259554124].

**Figure 6:**
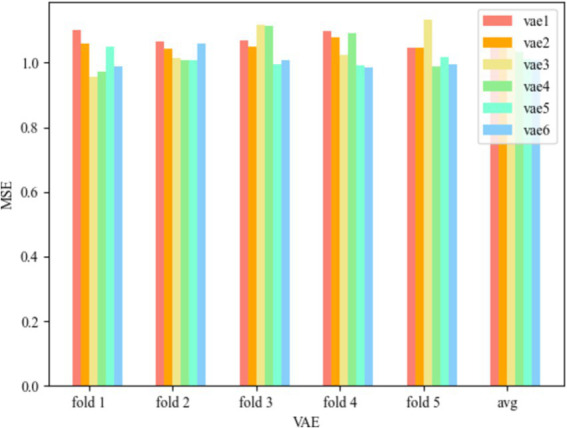
MSE of WR-CAE-OCSVM.

Figure 6 represents the MSE of WR-CAE-OCSVM for the six convolution kernel cases, and the Test MSE of the VAE model with convolution kernel (Wang et al., 2024; Liang et al., 2021) is 1.076472855, the Test MSE with convolution kernel (Liang et al., 2021; Chong et al., 2021) is 1.055950403, the Test MSE with convolution kernel (Chong et al., 2021; Mir et al., 2024) is 1.048900998, and the Test MSE of the VAE model with convolution kernel [16, 32] is 1.034770775, Test MSE for convolution kernel of [32, 64] is 1.012387657, and Test MSE for convolution kernel of [64, 128] is 1.007082856, which can be seen that the model error is minimum when the convolution kernel is [64, 128], and at this time Test MSE is 1.007082856, and the model error is maximum when the convolution kernel is (Wang et al., 2024; Liang et al., 2021), which is 1.007082856. At this time, the Test MSE is 1.076472855, and the error range is [1.007082856, 1.076472855].

Comparing the two models, it is obvious that the error of the model proposed in this paper is much lower than that of WR-VAE-OCSVM, and compared with the WR-VAE-OCSVM model, the minimum error is lower by 0.248944, which reduces it by 19.82%, and the accuracy of the WCOS model still performs better in a variety of test rounds, which verifies the validity and advancement of the model. To improve the detection accuracy of the model, the subsequent content will discuss the effects of hyperparameters in the model and find the optimal parameters, respectively.

#### 4.3.2 Hyperparameter determination

The modules that need to determine hyperparameters include the OCSVM module and the convolutional autoencoder module. The hyperparameters that the OCSVM module needs to determine are the control factor *C* and the kernel function *k*. Control factor Select *C* = 1 × 10^−4^, 1 × 10^−3^, 5 × 10^−3^, 1 × 10^−2^, 5 × 10^−2^ and 1 × 10^−1^ performance. Commonly used kernel function is linear function, polynomial function, radial basis function, the sigmoid function, formula by $k\left(h_{i},h_{j}\right)=\varphi {\left(h_{i}\right)}^{T}\varphi \left(h_{j}\right)$ is given by Equation 14:

(14) $$\begin{array}{l} k_{\mathrm{line}}\left(h_{i},h_{j}\right)={h_{i}}^{T}h_{j}\\ k_{\mathrm{poly}}\left(h_{i},h_{j}\right)={\left(a_{1}{h_{i}}^{T}h_{j}+b_{1}\right)}^{d}\\ k_{rbf}\left(h_{i},h_{j}\right)=\exp\left(-\frac{h_{i}-{h_{j}}^{2}}{\sigma^{2}}\right)\\ k_{\mathrm{sigmoid}}\left(h_{i},h_{j}\right)=\tanh\left(a_{2}{h_{i}}^{T}h_{j}+b_{2}\right) \end{array}$$

Where *a_1_* and *b_1_* are polynomial coefficients, *σ* are width parameters, and *a_2_* and *b_2_* are coefficients of the sigmoid function. What the convolutional self-coder module needs to determine is the convolutional kernel *i* and *j*. This article arranged the 6 kinds of combinations, respectively (*I* = 2, *j* = 4), (*I* = 4, *j* = 8), (*I* = 8, *j* = 16), (*I* = 16, *j* = 32), (*I* = 32, *j* = 64), (*I* = 64, *j* = 128). The results of each model are as follows.

**Figure 7:**
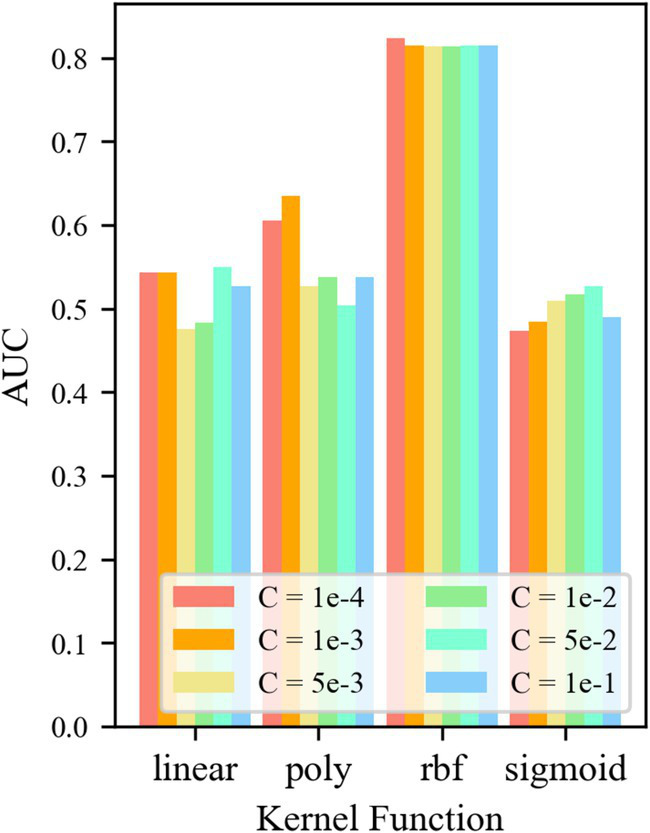
*AUC* at different parameters.

For the control group OCSVM, Figure 7 shows that the *AUC* value of the OCSVM model is higher only when the kernel function *k* = *k*_rbf_. When the kernel functions *k* = *k*_linear_, *k* = *k*_poly,_ and *k*_sigmoid_, the *AUC* values of the OCSVM model are lower. When the kernel function *k* = k_rbf_, the *AUC* value is less affected by the control factor C. When *k* = *k*_rbf_ and *C* = 1 × 10–4, the OCSVM model achieved the best performance in this task (*AUC* = 0.855).

**Figure 8:**
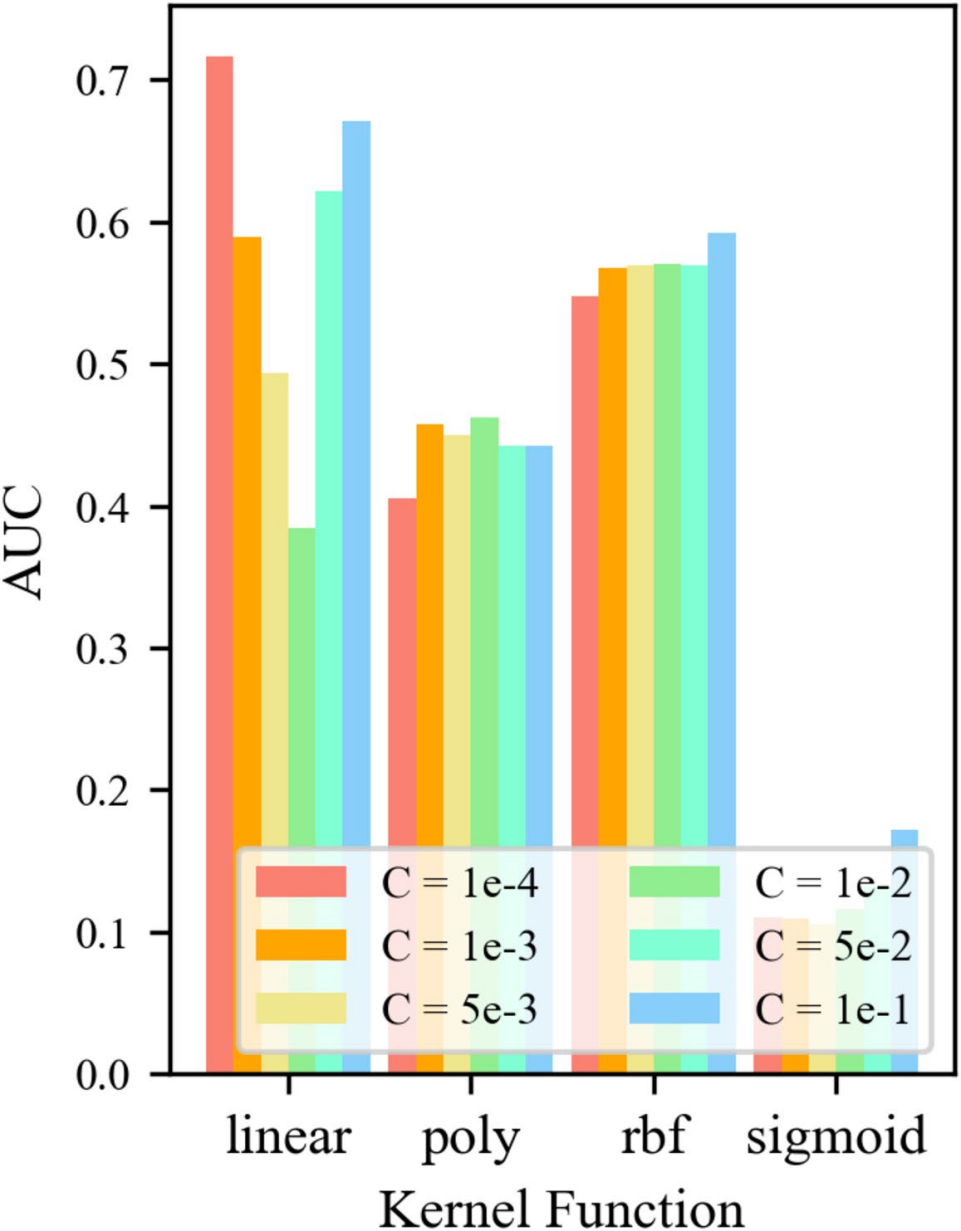
*AUC* for different parameters of TF24-OCSVM.

For control group No. 2 TF24-OCSVM, Figure 8 shows that when kernel functions *k* = *k*_rbf_ and *k*_linear_, the *AUC* value of the TF24-OCSVM model is higher. When the kernel functions *k* = *k*_poly_ and *k*_sigmoid_, the *AUC* value of the TF24-OCSVM model is low. When the kernel function *k* = *k*_rbf_, the *AUC* value is less affected by the control factor *C*. When the kernel function *k* = *k*_linear_, the *AUC* value has a greater influence on the control factor *C*. When *k* = *k*_linear_ and *C* = 1 × 10^−4^, the TF24-OCSVM model in this task achieved the best performance (*AUC* = 0.716).

**Figure 9:**
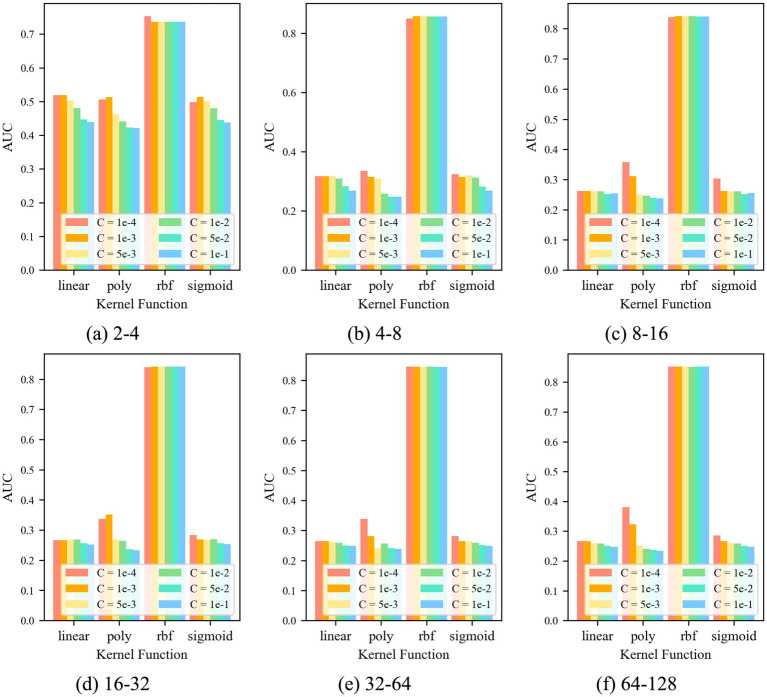
*AUC* of CAE-OCSVM with different parameters.

For the control group No. 3 CAE-OCSVM, the results are shown in Figure 9. Figure 9 shows that when *k* = *k*_rbf_, the *AUC* of the CAE-OCSVM model is significantly higher than the *AUC* of *k* = *k*_linear_, *k*_poly_, and *k*_sigmoid_. Therefore, the kernel function of the CAE-OCSVM model is set to *k*_rbf_. In this case, when the kernel is 2–4, the maximum *AUC* is 0.736 (*C* = 1 × 10^−3^). When the kernel is 4–8, the maximum *AUC* is 0.867 (*C* = 1 × 10^−3^). When the kernel is 8–16, the maximum *AUC* is 0.841 (*C* = 1 × 10^−3^)). When the Kernel is 16–32, the maximum *AUC* is 0.842 (*C* = 5 × 10^−2^). When the kernel number is 32–64, the maximum *AUC* is 0.846 (*C* = 1 × 10^−3^). When the kernel is 86–128, the maximum *AUC* is 0.852 (*C* = 1 × 10^−3^). Therefore, to achieve the best performance of the CAE-OCSVM model in this task, the kernel function is set to *k* = *k*_rbf_, the convolution kernel is set to 4–8, and the control factor is set to *C* = 1 × 10^−3^.

**Figure 10:**
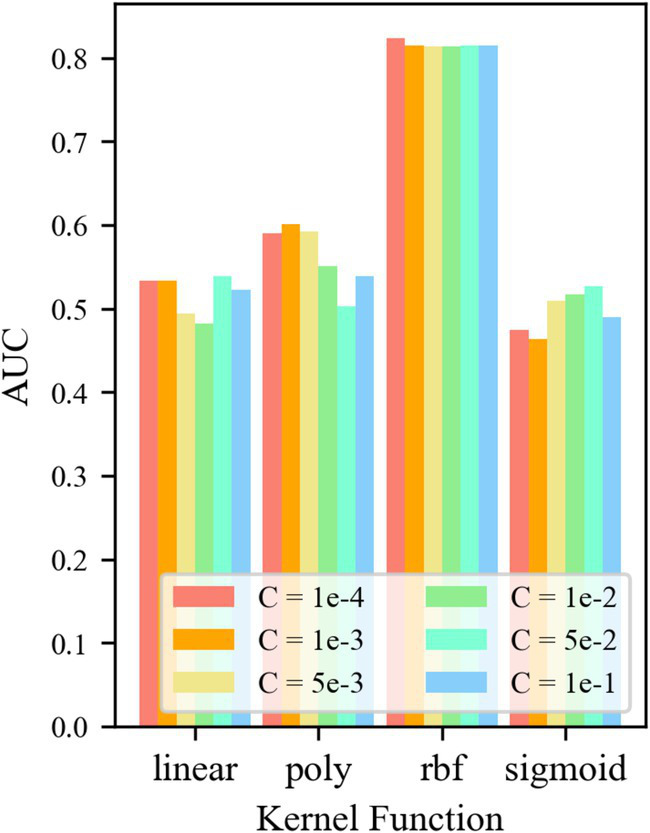
*AUC* at different parameters.

For control group No. 4 WR-OCSVM, the results are shown in Figure 10. Figure 10 shows that when kernel function *k* = *k*_rbf_, the minimum *AUC* value of the WR-OCSVM model reaches 0.814. When the kernel function *k* = *k*_linear_, the maximum *AUC* of the WR-OCSVM model reaches 0.538. When the kernel function *k* = *k*_poly_, the maximum *AUC* value of the WR-OCSVM model reaches 0.601. When the kernel function *k* = *k*_sigmoid_, the maximum *AUC* value of the WR-OCSVM model reaches 0.526. Therefore, when the kernel function *k* = *k*_rbf_, the lowest *AUC* value is significantly higher than the highest *AUC* value when the kernel function *k* = *k*_linear_、*k*_poly_、*k*_sigmoid_. When the kernel function *k* = *k*_rbf_ and the control factor *C* = 1 × 10^−4^, the *AUC* value is the highest, reaching 0.824. Therefore, to achieve the best performance of the WR-OCSVM model in this task, the kernel function is set to *k* = *k*_rbf_, and the control factor is set to *C* = 1 × 10^−4^.

**Figure 11:**
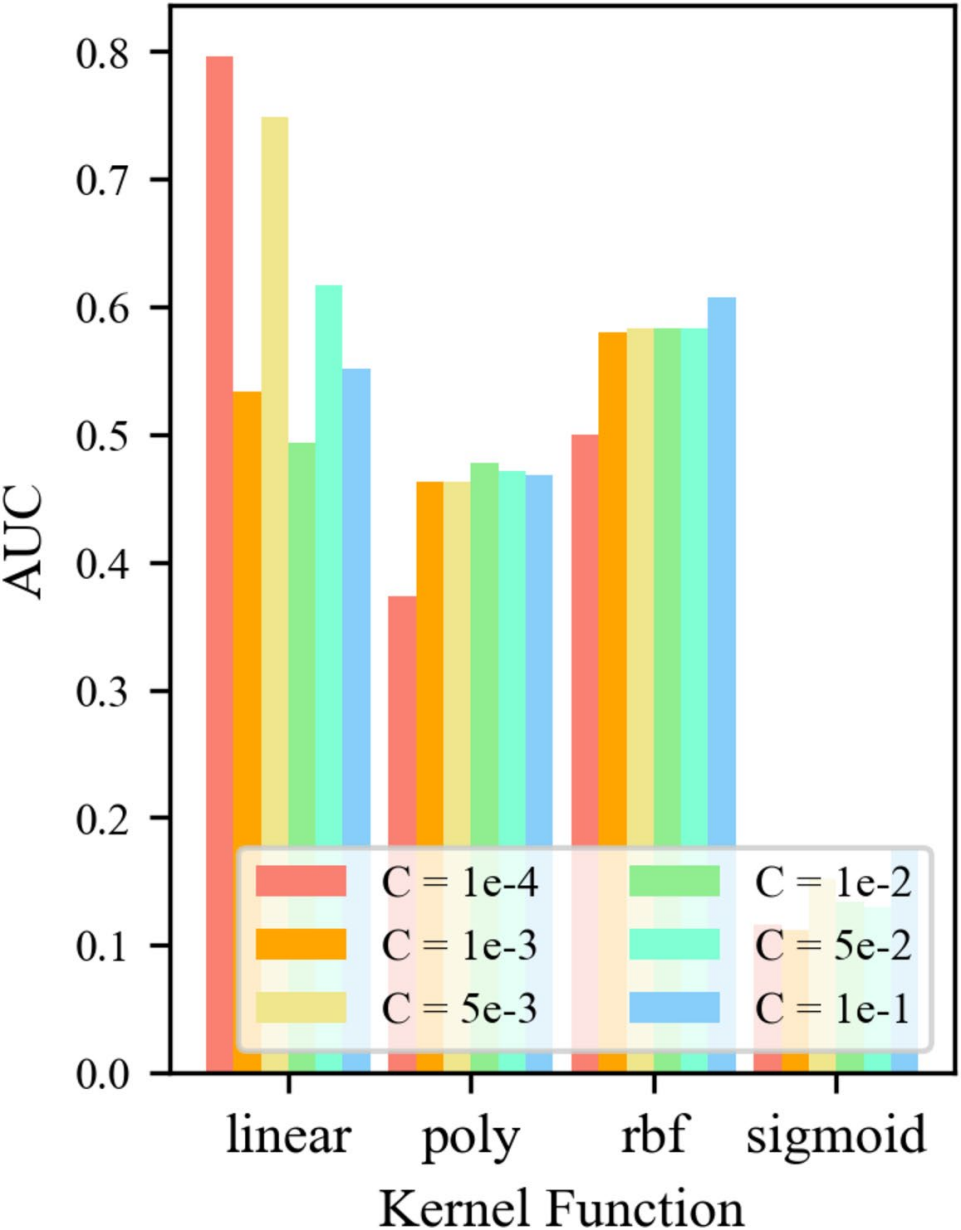
*AUC* at different parameters.

For the control group No. 5 WR-TF24-OCSVM, the results are shown in Figure 11. It is shown that when the kernel functions *k* = *k*_rbf_ and *k*_linear_, the *AUC* value of the TF24-OCSVM model is higher. When the kernel functions *k* = *k*_poly_ and *k*_sigmoid_, the *AUC* value of the TF24-OCSVM model is low. When the kernel function *k* = *k*_rbf_, the *AUC* value is less affected by the control factor *C*. When the kernel function k = klinear, the *AUC* value is greatly affected by the control factor *C*. When *k* = *k*_linear_ and *C* = 1 × 10^−4^, the TF24-OCSVM model in this task achieved the best performance (*AUC* = 0.796).

**Figure 12:**
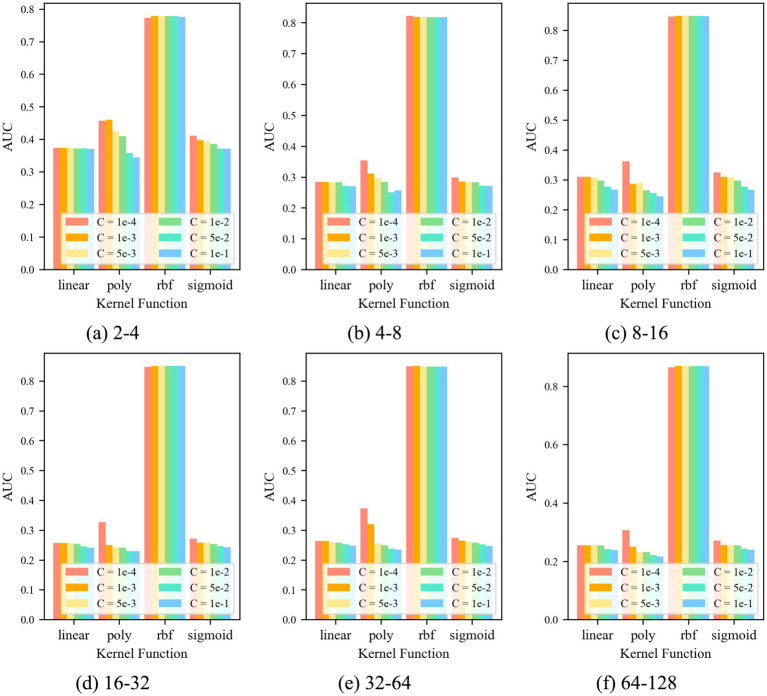
*AUC* at different parameters.

For the control group No. 6 WR-CAE-OCSVM, the results are shown in Figure 12: when *k* = *k*_rbf_, the *AUC* of the WR-CAE-OCSVM model is significantly higher than that when *k* = *k*_linear_, *k*_poly_, and *k*_sigmoid_. Therefore, the kernel function of the WR-CAE-OCSVM model is set to *k*_rbf_. In this case, when the kernel is 2–4, the maximum *AUC* is 0.779 (*C* = 1 × 10^−3^). When the kernel is 4–8, the maximum *AUC* is 0.822 (*C* = 1 × 10^−4^). When the kernel is 8–16, the maximum *AUC* is 0.848 (*C* = 1 × 10^−3^). When the kernel is 16–32, the maximum *AUC* is 0.850 (*C* = 1 × 10^−2^). When the kernel is 32–64, the maximum *AUC* is 0.851 (C = 1 × 103). When the kernel is 86–128, the maximum *AUC* is 0.870 (*C* = 1 × 10^−3^). Therefore, to achieve the best performance of the WRCAE-OCSVM model in this task, the kernel function is set to *k* = *k*_rbf_, the convolutional kernel is set to 64–128, and the control factor is set to *C* = 1 × 10^−3^.

In summary, the optimal hyperparameter Settings of the six groups of models are shown in Table 2.

**Table 2:** Hyperparameter setting.

Control group number	Control group model structure	Hyperparameter setting
1	OCSVM Model	OCSVM (rbf-0.0001)
2	TF24-OCSVM	OCSVM (linear-0.0001)
3	CAE- OCSVM	CAE-OCSVM (4-8-rbf-0.001)
4	WR-OCSVM	OCSVM (rbf-0.0001)
5	WR-TF24-OCSVM	OCSVM (linear-0.0001)
6	WR- CAE -OCSVM	CAE-OCSVM (64-128-rbf-0.001)

The table comprehensively shows the optimal hyperparameter settings of the six groups of models. The models are carefully tuned for different model structural characteristics, such as the OCSVM model kernel function is set to *k* = _krbf_ and the control factor *C* = 0.0001 in control group 1, the kernel function is set to *k* = k_linear_ and the control factor *C* = 0.0001 in control group 2, and the convolutional kernel is set to Liang et al. (2021) and Chong et al. (2021) for the CAE-OCSVM model in control group 3, the kernel function is set to Liang et al. (2021) and Chong et al. (2021), and the control factor *C* = 0.0001 in control group 4. the kernel function is set to *k* = k_rbf_, control factor *C* = 0.0001, as in control group 4 WR-OCSVM model kernel function is set to *k* = k_rbf_, control factor *C* = 0.0001, as in control group 5 WR-TF24-OCSVM model kernel function is set to *k* = k_linear_, control factor *C* = 0.0001, as in control group 6 WR- CAE -OCSVM model convolution kernel is set to [64, 128], the kernel function is set to *k* = k_rbf_, control factor *C* = 0.0001.

#### 4.3.3 Effectiveness of the developed framework

**Figure 13:**
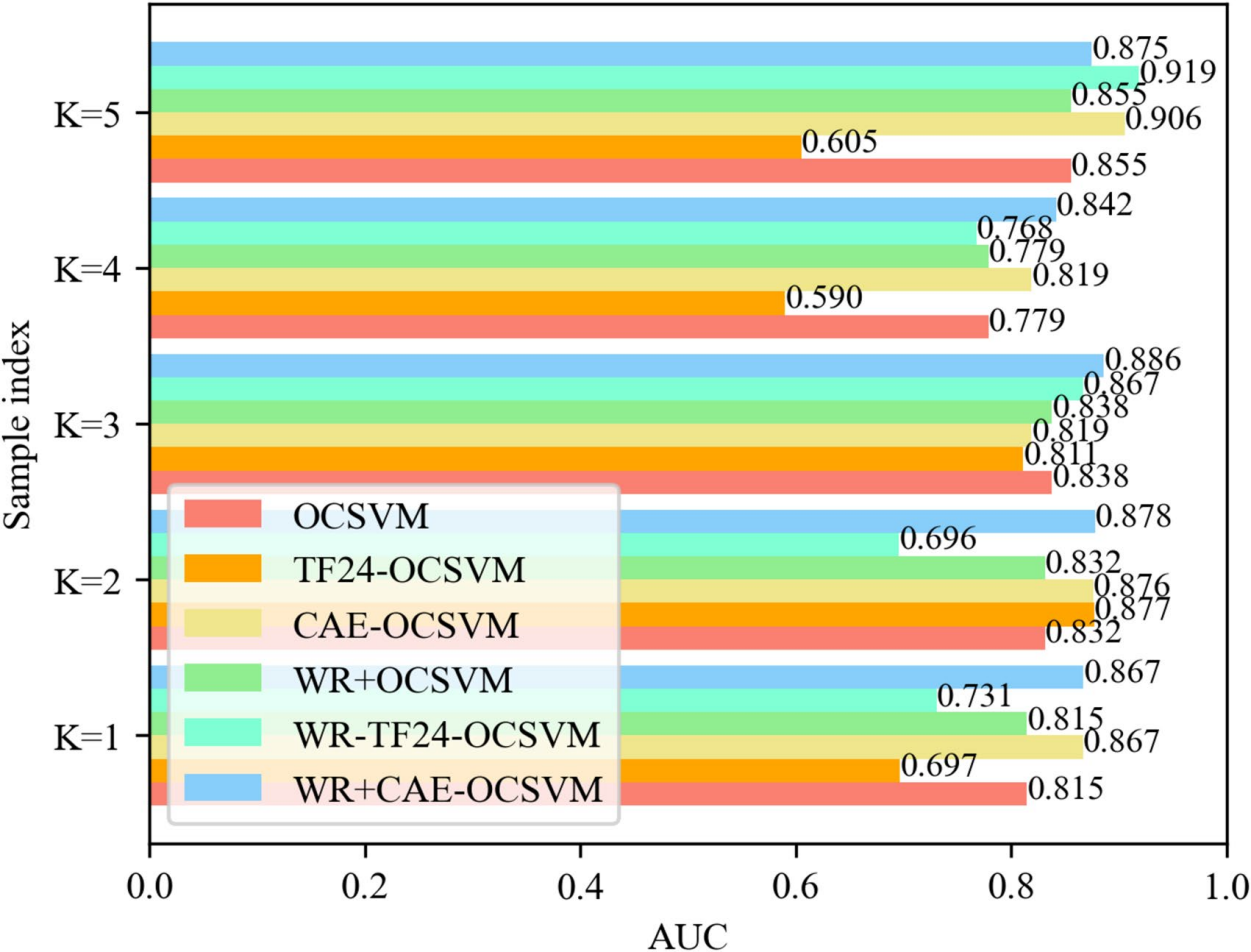
*AUC* for five-fold cross validation.

This paper established five groups of comparison experiments based on the cross-difference verification method to verify the validity. To remove the randomness of the neural network, each experiment was repeated 10 times. The experimental results are shown in Figure 13.

Figure 13 compares the *AUC* values of samples of six models OCSVM, TF24-OCSVM, CAE-OCSVM, WR-OCSVM, WR-TF24OCSVM, and WR-CAE-OCSVM under the best hyperparameters. It shows that the WR-CAE OCSVM has the highest *AUC* value in the samples with *K* = 1, 2, 3, and 4, reaching 0.867, 0.878, 0.886, 0.842, and 0.875, respectively.

Table 3 shows the Statistics of the mean, standard deviation, and variance of *AUC* of OCSVM, TF24-OCSVM, CAE-OCSVM, WR-OCSVM, WR-TF24-OCSVM and WR-CAE-OCSVM models, respectively.

**Table 3:** Mean, standard deviation, and variance of *AUC* for 6 models.

Model	*AUC* mean	*AUC* standard deviation	*AUC* variance
OCSVM	0.824	0.0290	0.0008
TF24-OCSVM	0.716	0.1260	0.0159
CAE- OCSVM	0.857	0.0378	0.0014
WR-OCSVM	0.824	0.0290	0.0008
WR-TF24-OCSVM	0.796	0.0938	0.0088
WR- CAE -OCSVM	0.870	0.0169	0.0003

Table 3 shows that the average AUC of WR-CAE-OCSVM is higher than that of WR-TF24-OCSVM (0.870–0.796)/ 0.870 × 100% = 8%. Similarly, the average AUC of WR-CAE-OCSVM is 5, 1, 18, and 5% higher than that of WR-OCSVM, CAE-OCSVM, TF24-OCSVM, and OCSVM, respectively. The above data reflect that WR-CAE-OCSVM has the best effect in this heart sound classification task.

Table 3 also shows that the AUC standard deviation of WR-CAE-OCSVM is lower than that of WR-TF24-OCSVM (0.0938–0.0169)/ 0.0938 × 100% = 82%. Similarly, the AUC standard deviation of WR-CAE-OCSVM is 42, 55, 87, and 42% lower than that of WROCSVM, CAE-OCSVM, TF24-OCSVM, and OCSVM, respectively. The AUC variance of WR-CAE-OCSVM is 97, 66, 80, 98, and 66% lower than that of WR-TF24-OCSVM, WR-OCSVM, CAE-OCSVM, TF24-OCSVM, and OCSVM, respectively. The above data reflect that the WR-CAE-OCSVM model has the best stability in this heart sound classification task.

#### 4.3.4 Anti-noise ability of the developed framework

**Figure 14:**
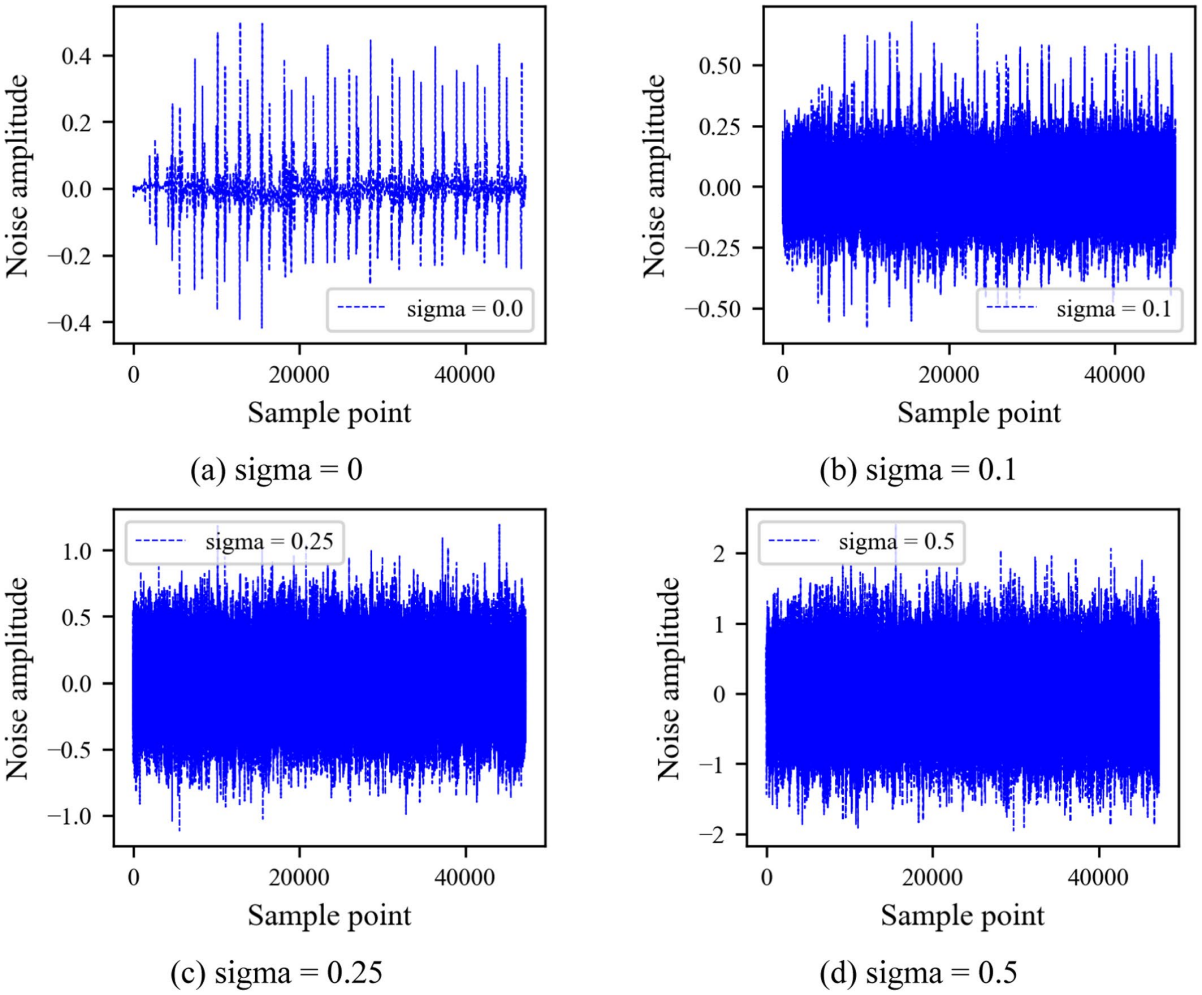
Four Gaussian noises with different sigma values.

To verify the anti-noise ability of the WR-CAE-OCSVM model in the classification of heart sounds, four groups of experiments were carried out, respectively. In the experiment, Gaussian noise with different standard variance (sigma) was added to heart sounds to simulate ambient noise. As shown in Figure 14, gaussian noise with four different sigma values is set.

**Figure 15:**
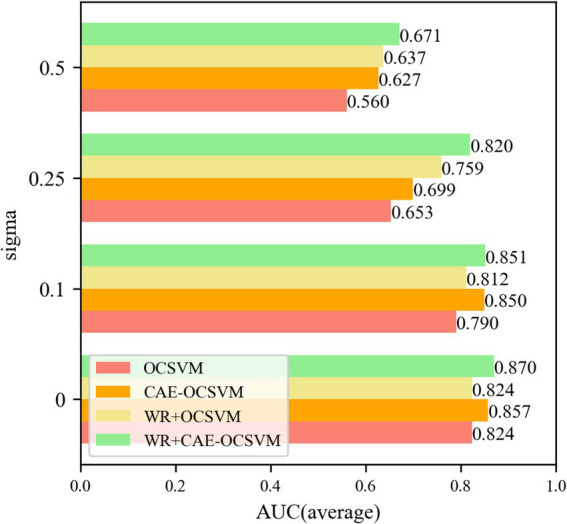
Sigma = 0, 0.1, 0.25, 0.5 anti-noise training *AUC*.

Five groups of experiments were established based on the cross-difference verification method, and four groups of noise were added to each group of experimental data. To eliminate the randomness of the neural network, each experiment was repeated 10 times, and the experimental results are shown in Figure 15.

**Figure 16:**
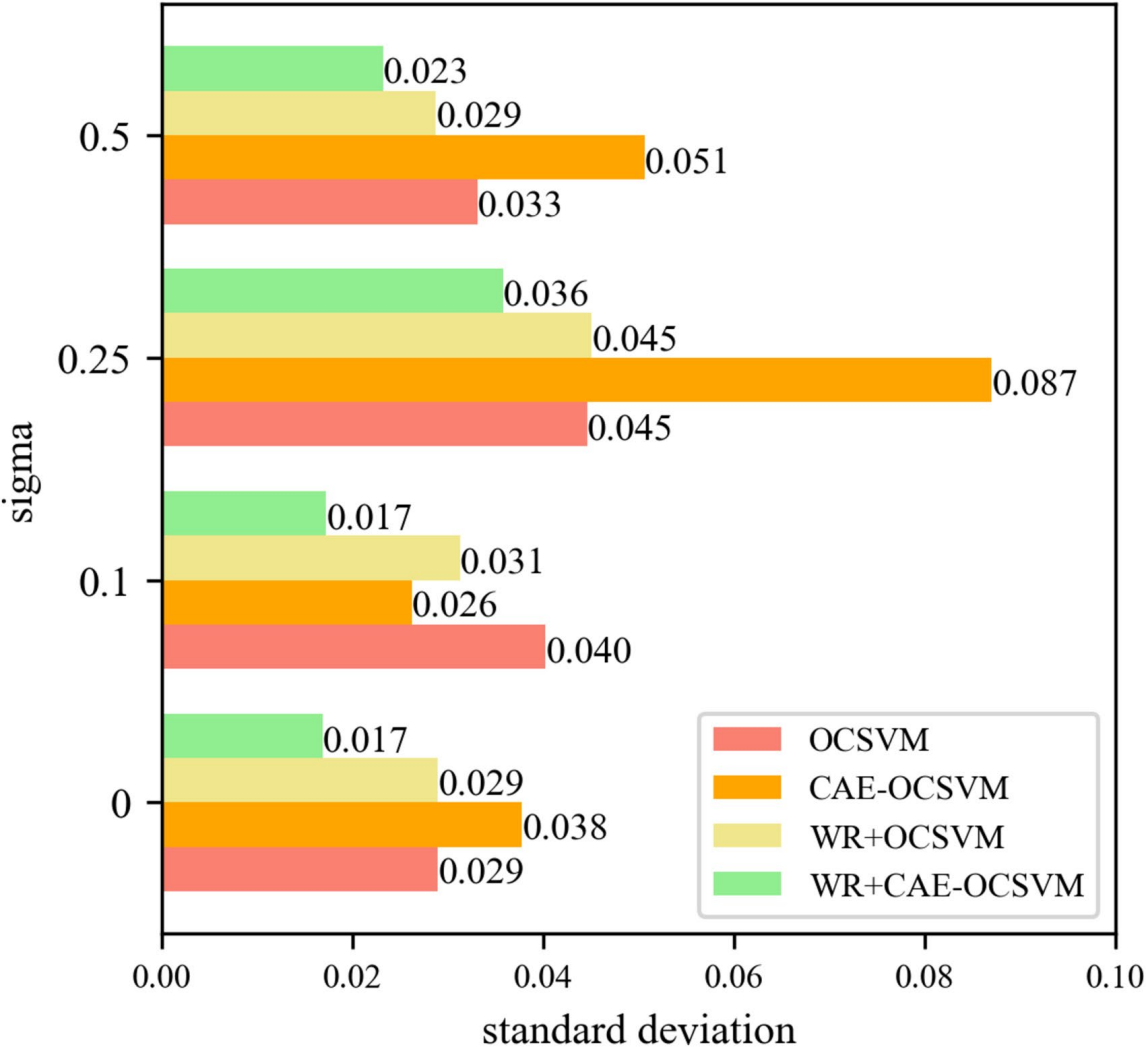
Sigma = 0, 0.1, 0.25, 0.5 anti-noise training *AUC*.

Figure 15 shows that the WR-CAE-OCSVM has the best classification effect in this task. When the noise of sigma = 0, the mean *AUC* of WR-CAE-OCSVM is higher than that of WR-OCSVM (0.870–0.824)/ 0.870 × 100% = 5.3%. The mean *AUC* of WR-CAE-OCSVM was 1.4 and 5.3% higher than that of CAE-OCSVM and OCSVM, respectively. When noise with sigma = 0.1, the mean *AUC* of the WR-CAE-OCSVM is 4.6, 0.2, and 7.2% higher than that of WR-OCSVM, CAEOCSVM and OCSVM, respectively. When noise with sigma = 0.25, the mean *AUC* of the WR-CAE-OCSVM is 7.4, 14.7, and 20.3% higher than that of WR-OCSVM, CAE-OCSVM and OCSVM, respectively. When noise with sigma = 0.5, the mean *AUC* of the WR-CAE-OCSVM is 5.0, 6.6, and 16.5% higher than that of WR-OCSVM, CAE-OCSVM, and OCSVM, respectively. To verify the stability of each model, Figure 16 shows the standard deviation of the *AUC* obtained from five sets of experiments.

Figure 16 shows that the WR-CAE-OCSVM model has the best stability in this task. When the noise of sigma = 0, the *AUC* standard deviation of WR-CAE-OCSVM is lower than the mean *AUC* of the WR-OCSVM (0.0290–0.0169)/0.0290 × 100% = 41.8%. The *AUC* standard deviation of the WR-CAE-OCSVM is 55.4 and 41.8% lower than that of CAE-OCSVM, and OCSVM, respectively. When the noise of sigma = 0.1, the *AUC* standard deviation of the WR-CAE-OCSVM is 44.9, 34.3, and 57.2% lower than that of WR-OCSVM, CAE-OCSVM, and OCSVM, respectively. When the noise of sigma = 0.25, the *AUC* standard deviation of the WR-CAE-OCSVM is 20.7, 58.9, and 19.8% lower than that of WR-OCSVM, CAE-OCSVM, and OCSVM, respectively. When the noise of sigma = 0.5, the *AUC* standard deviation of the WR-CAE-OCSVM is 19.2, 54.1, and 29.8% lower than that of WR-OCSVM, CAE-OCSVM and OCSVM, respectively.

## 5 Discussion and future perspectives

This paper presents a new semi-supervised anomaly detection method WCOS, which can work in both unsupervised and semi-supervised Settings. Experimental evaluations using real data sets show that WCOS is much more accurate than current anomaly detection methods and has greater noise resistance after adding different noise effects. In future work, we will improve the clustering process and anomaly measurement to make the detection more efficient and time-saving. Surveillance data is often uneven, so our future efforts will focus on discovering a suitable regularization technique that may further improve the stability of the anomaly detection model.

### 5.1 Advantages of the WCOS framework

In this paper, we propose a new semi-supervised anomaly detection method, WCOS, that can work in both unsupervised and semi-supervised settings. It has several significant advantages over other anomaly detection methods, firstly its semi-supervised nature is a key advantage, in the real world, obtaining a large amount of labeled data for heart sound anomaly detection is often both arduous and expensive, WCOS can effectively utilize both limited labeled data and a large amount of unlabeled data, by training a convolutional autoencoder with normal samples and using a one-class support vector machine, it can learn the normal heart sound’s inherent patterns and accurately differentiate abnormal heart sounds in the presence of sparse labeled abnormal samples, which greatly improves its practical applicability in the clinical setting.

Secondly, the integration of wavelet reconstruction provides significant noise suppression capability, heart sound signals are often contaminated by various noises, such as power line interference and baseline drift, wavelet reconstruction decomposes the signal into approximation coefficients and detail coefficients, and by eliminating the detail coefficients that mainly contain noise components, it effectively filters out the high-frequency noises while preserving the basic low-frequency trends and characteristics of heart sounds, and this denoised signal can be used as a subsequent This denoised signal can be used as a high-quality input for subsequent analysis, improving signal quality and the reliability of detection results.

The efficacy of the framework is further enhanced by the combination of a convolutional autoencoder and one classification support vector machine. The convolutional autoencoder has a convolutional and deconvolutional layer that extracts hierarchical abstract features from the heart sound data, which not only reduces the dimensionality of the data but also captures underlying patterns and correlations in the signal. One classification support vector machine then utilizes these learned features to establish accurate decision boundaries to distinguish between normal and abnormal heart sounds, and experimental results on real datasets fully demonstrate the effectiveness of WCOS.

### 5.2 Disadvantages and limitations

Despite its merits, the WCOS framework has certain drawbacks. The computational complexity during the training process is relatively high. The training of the convolutional autoencoder demands significant computational resources and time, particularly when handling large-scale heart sound datasets. The determination of optimal hyperparameters for the one-class support vector machine also requires extensive experimentation and fine-tuning, adding to the computational burden.

Moreover, while the model performs well in the current study focused on heart sound anomaly detection, its generalization to other types of medical signals or datasets with different characteristics may be limited. The model is trained and optimized based on the specific features and distributions of the heart sound data used in this research. When applied to other medical signal domains, such as electroencephalogram (EEG) or electromyogram (EMG) signals, or datasets with distinct statistical properties, its performance may decline due to differences in signal characteristics, noise patterns, and data distributions.

### 5.3 Future research directions

To address these limitations, future research efforts will be directed toward several aspects. In terms of computational efficiency, we plan to explore advanced model compression techniques. These techniques could involve pruning the redundant connections and neurons in the convolutional autoencoder without significantly sacrificing its performance. Additionally, we will investigate the application of parallel computing architectures to accelerate the training process. By distributing the computational tasks across multiple processors or computing devices, we can reduce the training time and make the framework more accessible for real-time or large-scale applications.

Regarding generalization, we intend to conduct more extensive cross-dataset validations. This will involve collaborating with other research institutions to access diverse medical signal datasets from different sources and populations. By evaluating the WCOS framework on these varied datasets, we can identify its strengths and weaknesses in different contexts and make necessary adjustments. Furthermore, we will explore the incorporation of domain adaptation algorithms. These algorithms can help the model adapt to new data domains by learning the transferable features and reducing the domain shift between the training and target datasets. This will enhance the model’s ability to generalize to a broader range of medical signal analysis tasks and contribute to the development of more robust and versatile anomaly detection methods in the medical field.

## Data Availability

The data analyzed in this study is subject to the following licenses/restrictions: data is unavailable due to privacy. Requests to access these datasets should be directed to Shuimiao Kang, 2022095018@cauc.edu.cn.
